# Characterization of treatment failure in HIV positive patients in the Colombian Caribbean region

**Published:** 2014-12-30

**Authors:** Juan Manuel De La Hoz, Laura Bolaño, Oriana Cárdenas, Robertulio González, José Sabbag, Lucy Palacio, Luz Marina Alonso, Homero San-Juan-Vergara, Guillermo Cervantes-Acosta

**Affiliations:** 1 Departamento de Medicina, Universidad del Norte, Barranquilla, Colombia.; 2 IPS QUIMIOSALUD. Barranquilla, Colombia; 3 Departamento de Salud Pública, Universidad del Norte, Barranquilla, Colombia

**Keywords:** HIV, virological failure, immunological failure, clinical failure, adherence, antiretroviral therapy

## Abstract

**Background:**

Treatment failure (TF) in patients receiving antiretroviral therapy against human immunodeficiency virus (HIV) is always a concern.

**Objective:**

To examine the correlates associated with TF in patients living in the Colombian Caribbean city of Barranquilla, an aspect that was poorly studied in this region.

**Methods:**

Treatment failure was evaluated in a cross-sectional study from virological, immunological and clinical standpoints.

**Results:**

It was established that 29.5% of patients under highly active antiretroviral therapy (ART) could be considered in TF. Among those, virological failure was most frequent (20.9%), followed by immunological- (14.0%) and clinical failure (4.7%). In patients showing lack of adherence to the treatment, the likelihood of suffering from TF and virogical failure were respectively increased by 6.67-fold and 12.19-fold, compared with patients showing good adherence. Although there was no statistically significant association, TF tended to be more frequent in young adults, in patients with low income and, low level of education. When ART regimens were compared, there was no apparent difference in TF between regimens based on non-nucleoside reverse transcriptase inhibitors and those based on protease inhibitors. This is very important in the context of recent ART strategies, such as early-initiated ART, aimed at achieving long-term infection control.

**Conclusions:**

Is confirmed the importance of treatment adherence to avoid TF and further highlights the importance of educating HIV-infected patients in all parts of the world, especially those individuals with a lower socio-economic status.

## Abstract (es)

**Objetivos:**

El fracaso terapéutico (FT) en pacientes que están recibiendo terapia antirretroviral contra el Virus de la Inmunodeficiencia Humana (VIH) ha sido siempre preocupante.

**Objetivo:**

Examinar las variables asociadas con el fracaso terapéutico, un problema que ha sido pobremente estudiado en la región, en pacientes que habitan en Barranquilla, ciudad del Caribe colombiano.

**Métodos:**

El fracaso terapéutico fue evaluado en un estudio transversal desde los puntos de vista virológico, inmunológico y clínico.

**Resultados:**

Se determinó que el 29.5% de los pacientes bajo terapia antirretroviral (TAR) de gran actividad podría considerase en fracaso terapéutico. El fracaso virológico fue el más frecuente (20.9%), seguido por el inmunológico (14.0%) y el clínico (4.7%). En pacientes que mostraron falta de adherencia al tratamiento, las posibilidades de presentar un fracaso terapéutico y virológico fue mayor, 6.67 y 12.19 veces, respectivamente, comparados con pacientes que mostraron buena adherencia. Aun cuando no hubo una asociación estadística significativa, el fracaso terapéutico tendió a ser más frecuente en adultos jóvenes, en pacientes con bajos ingresos y bajo nivel de educación. Cuando se compararon diferentes regímenes de TAR no hubo una diferencia aparente entre el fracaso terapéutico en los regímenes basados en inhibidores no nucleósidos de la transcriptasa inversa y en aquellos basados en inhibidores de la proteasa. Esto es muy importante en el contexto de recientes estrategias de TAR, como es el caso de las iniciadas en forma temprana con el fin de lograr el control de la infección a largo plazo.

**Conclusiones:**

Se confirma la importancia de la adherencia al tratamiento para evitar el fracaso terapéutico y la importancia de educar a los pacientes infectados con el VIH, especialmente aquellos individuos con un nivel socioeconómico bajo.

## Introduction

Despite the drawbacks in the development of a successful vaccine against HIV 1, the development of therapeutic regimens using drug combinations has significantly increased survival and reduced HIV-associated morbidities in HIV-infected individuals. These antiretroviral therapies (ART) interfere with viral replication which results in slowing the natural course of the infection 2. However, during the course of the treatment, some HIV-infected individuals may not respond as expected, which has been defined as treatment failure (TF) 3.

Treatment failure may be classified as virological, immunological and clinical failure. Virological failure is defined as an increase of more than 1,000 copies of RNA /mL or the re-appearance of a signal after a period during which it has been undetectable. The World Health Organization (WHO) defines immunological failure as a decline trend in CD4 T cells count despite 6 months of treatment, or a failure to increase the CD4 T cells counts above 100 cells/mm^3^ after 12 months of treatment. Clinical failure is defined by WHO as the appearance of any morbidity associated to category 4, despite 6 months of treatment 4 ^-^ 6.

Preventing the development of TF is critical, since such condition imposes a regimen change to successfully treat HIV-infected individuals experiencing TF. Such regimen change would mean an increase in treatment-associated costs 6. Consequently, it is important to identify those factors associated with the appearance of TF, including the evaluation of those ART regimens used in treating the condition. Erroneous decisions regarding ART regimens would mean a continuous administration of either an ineffective drug, or of a suboptimal dose of an otherwise effective drug, with the risk of selecting resistance-associated mutations 7 ^-^ 9.

According to WHO statistics, 170,000 HIV-infected people have been diagnosed in Colombia. Among those, 21,000 people are receiving ART, which only represents 39% of the 54,000 people considered to require therapy 10. The presence of TF in Colombia is suspected but the real dimension of the problem is unknown. The group of Diazgranados *et al*. 11, reported predictors of optimal virological response present in a sample of HIV-infected in Colombia, but such report only analyzed 27 individuals from Barranquilla. In the current study the common types of TF and the respective factors associated to such failure in individuals in ART was examined in a much larger cohort of individuals living in Barranquilla. 

Even though there was no statistically significant association, sociodemographic characteristics showed a tendency of TF to be associated with younger adults, low income, or low level of education; while being in middle or upper socio-economic class was prone to be a protective factor. When antiretroviral therapies (ART) regimens were compared in the context of treatment failure, there was no difference between those based on non-nucleoside reverse transcriptase inhibitors with those based on protease inhibitors.

## Materials and Methods

###  Study design

A cross-sectional study was conducted to characterize the response of HIV-infected patients under treatment enrolled in one of the leading clinical centers at Barranquilla for at least two years. After obtaining IRB's approval, the clinical registries of 385 HIV-infected patients were reviewed, from which 129 patients were chosen based on the following criteria: aged older than 18 years, who had complete records in terms of both CD4 count and viral load (2 times a year), and who have been followed clinically for at least 1 year. From HIV-infected people enrolled in the clinic, those that were pregnant or non-treated due to good immunological standing were excluded from the study.

From each patient, socio-demographical characteristics that were taken into account for the study included age, gender, socio-economical class, level of education. On the other hand, the regimen treatment and its duration in years were also recorded. The regimen treatment was classified according to those using non-nucleoside, reverse transcriptase inhibitors (e.g. Efavirenz) or protease inhibitors (e.g. Lopinavir). 

Adherence to treatment was measured by establishing the ratio between the amounts of drug that were ingested to the quantity of the medication that was provided by the clinical center. The cut-off for an acceptable adherence ratio was estimated to be ≥80%.

CD4 count and viral load as well as patient's clinical status were used to determine treatment failure and the respective classification (virological, immunological and clinical failure). (TruCount CD4 BD and NucliSENS EasyQ HIV-1 2.0). 

Patient's data were systematically recovered from the clinical registry and stored in a way that erases any personal identifier. Such data were analyzed through descriptive statistics using the statistical package SPSS^(r)^. Such evaluation was conducted using bivariate analysis through contingency tables 2x2, in which each variable was crossed with each one of the TF categories. The independence of the different variables was confirmed and a chi-square test was used. In those cases where a value recorded in a cell was below than 5.0, a Fisher test was used. A *p* <0.05 was chosen as statistical significance.

## Results

Seventy-eight patients were excluded. Among them, 27 were excluded due to incomplete clinical registries; 19 due to an age lower than 18 years old; 30 due to a treatment period less than 1 year; 1 pregnant woman at the time of the study; and 1 non-treated patient due to good immunological status. 

Among those 129 patients that complied with the inclusion criteria: 50% were younger than 39.6 years; 72.9% were males; 69.8% belong to the lowest levels of socio-economic status (1 and 2), 27.1% was classified as middle class (3 and 4), and 3.1% was at the highest socio-economic status (5 and 6); and only 45.0% reached a degree in a technical / vocational school, while 55% did not finish high school (Table 1).

**Table 1.**
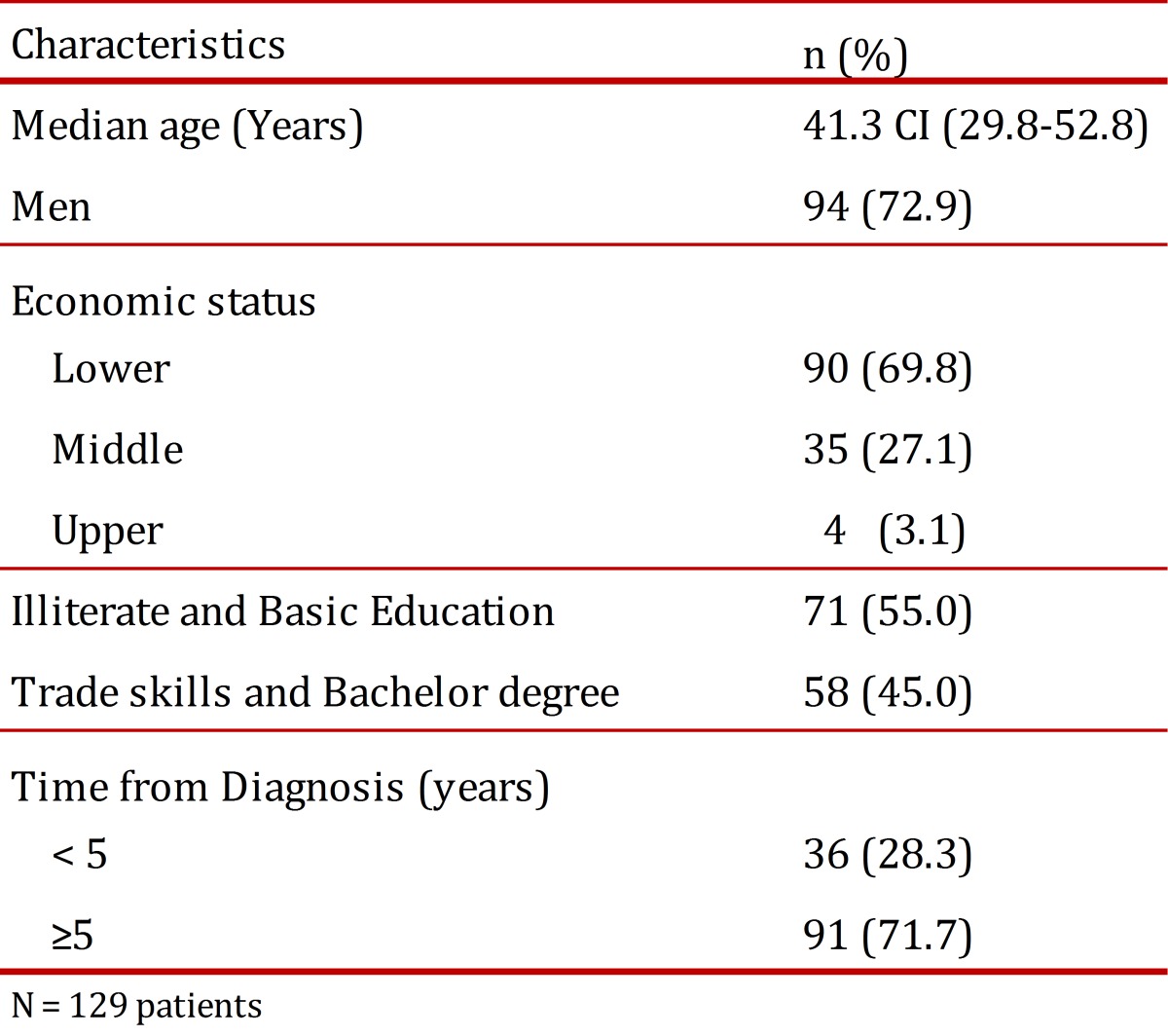
Sociodemographic characteristics of the patients enrolled in the study. Information was collected from patients' clinical registries who previously accepted to become part of the study under informed consent.

Therapeutic failure was identified in 38 patients (29.5% of the studied patients); among them, 27 were male (71.0%) and 11 were female (29.0%). Some of the patients with therapeutic failure simultaneously had more than one kind of failure (e.g. virological and immunological at the same time). Virological failure was the most frequent event (20.9%) followed by the immunological (14.0%) and the clinical failure (4.7%).

### Adherence

Approximately, 92% of the patients demonstrated an acceptable adherence to the treatment regimen. As expected, 70% of those patients with non-acceptable adherence suffered therapeutic failure; virological failure being the event most frequently recorded (70%) followed by immunological failure (30%). None of these patients suffered clinical failure. Bivariate models showed that those patients with non-acceptable adherence were 6.67 more likely to experience therapeutic failure than those who had good adherence (OR: 6.67; CI: 1.61-27.54; *p*= 0.007) (Table 2); in the same way, those with non-acceptable adherence were 12.19 more likely to suffer virological failure (OR: 12.19; CI: 2.49-66.8; *p*< 0.001) (Table 3).

**Table 2.**
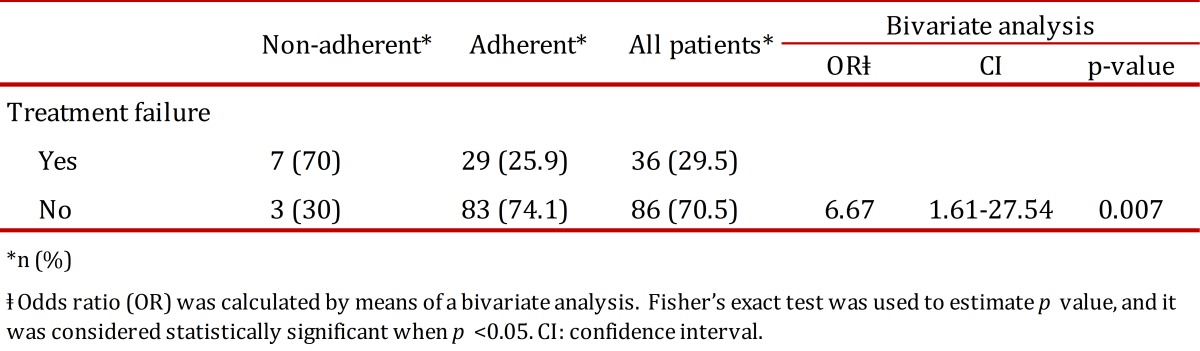
Outcome according to the treatment adherence. Patients who suffered treatment failure were sorted by antiretroviral therapies adherence.

**Table 3.**
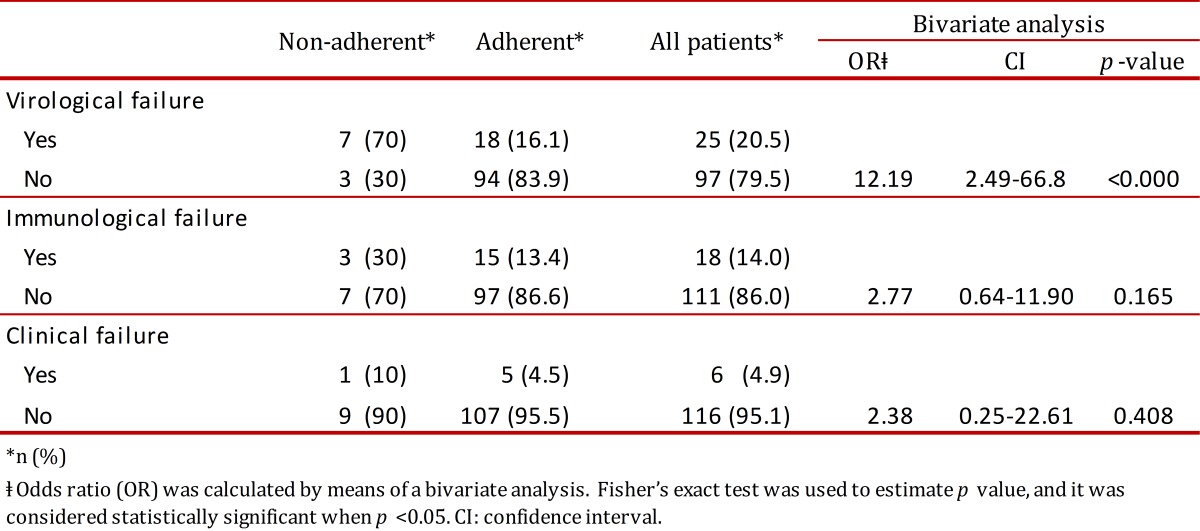
Distribution of the patients on virological, immunological and clinical failure classified according to the ART adherence. Patients who suffered treatment failure were sorted by the type of failure and ART adherence.

### Socio-demographical factors

Most of the events of therapeutic failure were seen in those patients that were in the lower rank of the socio-economic status (34.4%) compared with those seen in patients belonging to middle or upper rank of the socio-economic class (18.0%). However, such correlation did not reach statistical significance (OR: 2.40; CI: 0.95-6.06; *p*= 0.059). Neither was a statistical association found when the following variables were studied in the context of treatment failure: age (OR: 1.67; CI: 0.77-3.64; *p*= 0.190), gender (OR: 0.88; CI: 0.35-2.22; *p*= 0.76), and education (OR: 1.37; CI: 0.63-2.96; *p*= 0.41).

### Treatment regimen

In the population under study, the following treatment regimens were ranked from the most to the least used: (1) Efavirenz + Lamivudine + Zidovudine (29.5%), (2) Lamivudine + Zidovudine + Ritonavir + Lopinavir (14.0%), (3) Abacavir + Lamivudine + Ritonavir + Lopinavir (5.4%), (4) Abacavir + Lamivudine + Efavirenz (5.4%), and (5) Atazanavir + Zidovudine + Ritonavir + Lopinavir (4.7%). A large share of individual treatment regimens was assigned to few patients; overall they represent 41.1% of the 129 patients enrolled for this study. The different sets of antiretroviral therapies provided to the patients were grouped in two types: as those based on non-nucleoside reverse transcriptase inhibitors and those based on protease inhibitors. No association was found between the type of treatment regimen and the occurrence of treatment failure. (OR: 0.44; CI: 0.13-1.44; *p*= 0.28) (Table 4).

**Table 4.**
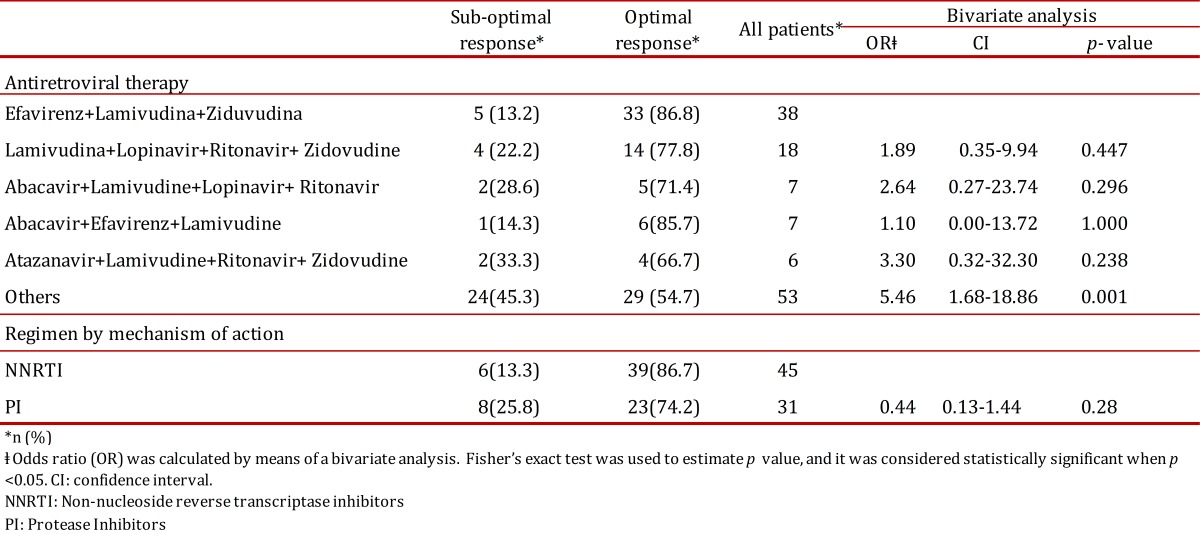
Outcome according to the type of treatment regimen. Regimen treatments were classified according to its frequency of administration and to antiretroviral mechanism of action.

## Discussion

The current study was conducted to determine which kind of treatment failures were more frequently found in a representative sample of HIV-infected patients living in Barranquilla, a major Caribbean coastal city in Colombia. On the other hand, factors recorded in the clinical registries were examined for their association to treatment failure and to its respective classes. It was found that 29% of those patients receiving antiretroviral therapy suffered treatment failure. Such percentage was close to previous percentage found in other countries with a gross domestic product similar to Colombia 12 ^,^ 13. However, studies conducted in Latin American countries showed a great disparity in terms of percentage of treatment failures 14 ^,^ 15. 

Although it cannot be considered statistically significant, the current study also indicated a higher percentage of treatment failure in male patients. Previous studies conducted in China also highlighted male gender as a significant risk factor for high mortality in the context of ART resistance 16. A study in Nigeria showed the same trend regarding male gender being more prone to suffer from treatment failure to ART 17. The fact that HIV-infected males may be at risk for treatment failure emphasizes the need to identify those associated factors explaining such a result, and consequently to tailor health strategies to this population.

Virological failure was the most frequent treatment failure found in the studied population, around 20.9% of the HIV-infected people under treatment, which was similar to previous reports 18. However, a retrospective study conducted in United States found a higher percentage of virological failure 19, but such disparity might be explained by certain particularities and hidden variables resulting from the composition and intrinsic dynamics of both populations with a very strong component of white and Afro-American race in the American study while patients in the current study predominantly consisted of Hispanics. On the other hand, it is plausible that different kinds of circulating virus groups in United States may result in a higher risk of treatment failure compared with what has been found for Colombia, which different studies point to just one circulating group. In clinical decision making, virological failure is by far the single most important criterion used to change treatment course. However, the fact that lack of adherence was associated to virological failure in the current study, as also demonstrated in other instances, implies that it is better to resolve the adherence issue and its causes before changing ART regimen. A low adherence to treatment is associated to a low serum concentration of the antiretrovirals and implies the risk of not having the antiviral optimum concentration to curb virus production 18.

Although there was not significant association between socio-demographical aspects, such as economic status and education, with treatment failure, it was clearly observed that most of the HIV-infected people belonged to the lower socio-economic status (69.8%). In addition, most of them had not finished high school (55.0%). The combination of these 2 factors has been previously reported to be associated with virus transmission in a community with lacks an adequate education and information regarding the infection 20. 

When antiretroviral therapies (ART) regimens were compared in the context of treatment failure, there was no difference between those based on non-nucleoside reverse transcriptase inhibitors with those based on protease inhibitors. This fact is very important in the context of the recent ART strategies, such as early-initiated ART, aiming at achieving long-term infection control and the search for a functional HIV cure. When those antiretroviral regimens that were only provided to few patients are grouped, these patients were found to be 5.46 more likely to suffer from treatment failure than those treated with the newest antiretroviral regimen Efavirenz + Lamivudine + Zidovudine (Table 4). In the former group the patients had the longest disease course, and they were previously subjected to different treatment regimens, which would suggest the accumulation of several mutations that confer HIV resistance to the antiretrovirals. The early detection of treatment failure may help limiting the accumulation of such mutations that potentially could impair the next regimen. Conducting resistance tests in those patients suffering from virological failure would help to decide the need of changing to a different regimen 21. One important fact to keep in mind is the host genetic framework since certain drug-metabolizing enzymes may result in a suboptimal serum concentration which produces either adverse side effects or poor viral control 22 ^,^ 23.

Overall, these results have concurred about the importance of learning about treatment failure causes to properly treat HIV-infected individuals in a large city in Colombia, a place where the limited resources need such vigilance to help us taking the proper clinical decisions.
